# Cortisol reactivity to psychosocial stress is greater in sexual risk takers

**DOI:** 10.1080/21642850.2014.889571

**Published:** 2014-02-20

**Authors:** Claire Harrison, Joanne M. Ratcliffe, Melanie Mitchell, Michael A. Smith

**Affiliations:** ^a^ Department of Psychology, Northumbria University, Newcastle upon Tyne, UK

**Keywords:** HPA axis, cortisol, stress reactivity, sexual risk taking

## Abstract

Several studies have reported an association between deviant behaviour and cortisol reactivity to stress. However, relatively few studies have investigated the relationship between psychobiological stress reactivity and sexual risk-taking behaviours. In this study, cortisol reactivity to the Trier Social Stress Test (TSST) was measured in 26 healthy young adults prior to the administration of a sexual health and behaviour questionnaire. The cortisol response to the TSST was greater in those individuals who reported that at least one of their previous two sexual partners was someone whom they had just met. Results are discussed in the context of a model which suggests that early life stress dysregulates the hypothalamic–pituitary–adrenal axis and increases the likelihood of later life risk-taking behaviour. The findings have implications in terms of improving our understanding of psychobiological factors which predispose individuals to engage in adverse sexual health behaviours.

## 1. Introduction

The hypothalamic–pituitary–adrenal (HPA) axis is one of the primary neuroendocrine pathways involved in the psychobiological stress response. In response to certain stressors, corticotropin releasing hormone is secreted from the hypothalamus, which stimulates the release of adrenocorticotropin hormone from the anterior pituitary. In turn, this stimulates the release of the final HPA effector hormone, cortisol. Cortisol plays an important role in preparing the body to cope with the challenges posed by stressors. It is involved in the mobilisation of energy reserves via its regulatory effects on blood glucose and by temporarily dampening the activity of physiological pathways which are not directly involved in dealing with the threat and restoring homeostasis, such as the immune system. Further, HPA regulation is maintained via the negative feedback of cortisol on the hypothalamus (Sapolsky, Romero, & Munck, 2000). Cortisol reactivity to acute stress can be measured in the laboratory, with those laboratory stressors which are characterised by a high level of uncontrollability and socio-evaluative threat being most amenable to increases in salivary cortisol (Dickerson & Kemeny, 2004).

The cortisol response to stress is an important biological process which partially facilitates the behavioural ‘fight or flight’ response to acute stress exposure (McEwen, 2000). However, it is now clear that there are vast inter-individual differences in the cortisol response to stress. Dysregulation of the cortisol response to stress, characterised by relative hyper- or hypo-secretion of cortisol in response to acute stress, prolonged cortisol exposure or poor habituation of the HPA axis to repeated exposure to routine events has been linked to adverse environmental influences, including low socioeconomic status (SES; Cohen, Doyle, & Baum, 2006a; Cohen et al., 2006b; Lupien, King, Meaney, & McEwen, 2001), poor maternal mental health (Van den Bergh, Van Caslster, Smits, Van Huffel, & Lagae, 2008) and abuse (Tarullo & Gunnar, 2006) during the developmental period. Exposure to early life stress during sensitive critical periods of biological development has also been linked with adverse antisocial behaviour later in the lifespan (Ehlert, 2013; Susman, 2006).

A number of studies have sought to investigate the relationship between deviant behaviour and HPA (dys)function. Basal hyposecretion of cortisol is associated with a range of antisocial behaviours including aggression (Platje et al., 2013) and violence (Brewer-Smyth, Burgess, & Shults, 2004). However, in terms of the relationship between antisocial behaviour and HPA reactivity, results are relatively more mixed. While aggression (Gordis, Granger, Susman, & Trickett, 2006) and conduct disorder (Fairchild et al., 2008) have been associated with attenuated cortisol responses to acute stress, a previous study reported that cortisol reactivity to psychosocial evaluation was associated with antisocial behaviour and rule-breaking in later developing boys (Susman et al., 2010). A further study failed to find a relationship between cortisol reactivity and externalising behaviour (Alink et al., 2008).

While a number of studies have considered the relationship between HPA reactivity and antisocial behaviour, relatively few studies have sought to investigate the relationship between the cortisol response to acute stress and sexual risk taking. Sexual risk taking is a cluster of deviant behaviours which can be considered deviant due to the increased risk from engaging in these behaviours of unplanned pregnancy or sexually transmitted infections (STIs; Hoyle, Fejfar, & Miller, 2000). Sexual risk-taking behaviours, therefore, vary between individuals depending on a number of factors such as pregnancy intentions and exclusivity of both partners, but generally include such activities as intercourse without the use of condoms and/or other methods of contraception, as well as early age at first sexual intercourse (Tripp & Viner, 2005). In one previous study, which investigated the relationship between acute stress reactivity and sexual risk-taking behaviour, young adults who showed the greatest cortisol response to the stressor reported having relatively fewer lifetime intercourse partners, but also less frequent condom use (Halpern, Campbell, Agnew, Thompson, & Udry, 2002). In a further study, females reporting an earlier age at first sexual intercourse exhibited an attenuated cortisol response to the Trier Social Stress Test (TSST; Brody, 2002). Thus, the findings from previous research, which have investigated the relationship between cortisol reactivity and sexual risk taking, have been mixed with one risk-taking behaviour (less frequent condom use) being associated with greater cortisol reactivity to acute stress, but two other risky behaviours (early age at first intercourse and a greater number of lifetime sexual partners) being associated with an attenuated cortisol response. Further, the conclusions which can be drawn from one of these previous two studies (Brody, 2002) are limited by the fact that half of the participants were administered high-dose ascorbic acid supplements, while this study also neglected to collect data on sexual risk-taking variables other than age at first sexual intercourse, which limits the extension of the findings to other risk-taking behaviours.

In this study, we sought to investigate the relationship between cortisol reactivity to the TSST and self-reported sexual risk-taking behaviour in young adults. In addition to age at first sexual intercourse, number of lifetime sexual partners (both continuous variables), condom use and contraction of STIs (both dichotomous variables), we also included a dichotomous outcome variable in which respondents were asked whether either of their two most recent sexual encounters were with someone whom they had just met. We were particularly interested to ascertain whether patterns of cortisol reactivity might be different for individuals engaging in different risky sexual behaviours, as predicated by previous findings in the literature (Brody, 2002; Halpern et al., 2002).

## 2. Method

### 2.1. Participants

A total of 30 participants ranging between 19 and 25 years of age were initially recruited to the study. However, an insufficient quantity of saliva was obtained for analysis at one or more sampling points for four participants. Thus, complete data were available for the analysis of 26 participants (12 males; *M*_age_ = 22.4, SD_age_ = 1.7). Participants were recruited as a part of an opportunity sample from around the North East of England. The study was advertised via emails sent to University distribution lists and by word-of-mouth. It was required that all participants were aged 18–25 and were exclusively heterosexual. Participants could not participate if they had ever received a diagnosis of clinical depression, had an endocrine disorder, were pregnant, used steroid medication or reported being ill on the day of testing. None of the participants who were recruited to the study fulfilled any of the exclusion criteria, and thus data from all participants were used in the analyses reported here. Participants received no reimbursement for taking part in the study.

### 2.2. Materials

#### 2.2.1. Trier Social Stress Test

The TSST (Kirschbaum, Pirke, & Hellhammer, 1993) is a well-established stress reactivity task that has shown to be effective in inducing increases in salivary cortisol levels (Dickerson & Kemeny, 2004). The task comprised a five-minute public speaking task in front of a panel of three people, all of whom were unknown to the participant. Participants were asked to convince the panel in their speech that they are the best candidate for their ‘dream job’. If participants completed their speech before the five minutes elapsed, they were prompted to continue speaking until they were not able to say anything else on the topic, at which point the researchers posed questions to the participant relating to their chosen job to prompt them to continue speaking. Prior to the public speaking task, participants were allowed a five-minute period to prepare their speech. Following the completion of the public speaking phase, participants completed a five-minute unprepared mental arithmetic task which was also performed in front of the panel of three people. This task required participants to serially subtract 17 from 2038. If a mistake was made, participants were prompted to start again, and any questions from the participants regarding their performance or the amount of time left were ignored. Participants were told that the task was also being video recorded throughout to increase the perception of social evaluation. While a video camera was trained on the participant throughout the task, no such recording was made.

#### 2.2.2. Salivary cortisol

Saliva samples were taken from participants at five intervals throughout the TSST: baseline (0 minutes), after instructions were given (5 minutes), after the preparation period (10 minutes), immediately after the task (20 minutes) and following a 20-minute rest period (40 minutes). Participants chewed on the cotton swab of a Salivette tube (Sarstedt, Nümbrecht, Germany) for 1–2 minutes before returning this to the researcher. All saliva samples were then stored in a freezer at −20°C. At the time of analysis, they were thawed and centrifuged at 3000 rpm for 15 minutes to extract the saliva from the cotton swab. Salivary cortisol was then quantified for each sample by luminescence immunoassay (Salimetrics Europe, Newmarket, UK) conducted in accordance with the manufacturer's instructions. Intra- and inter-assay coefficients of variation were less than 10%.

#### 2.2.3. Sexual behaviour questionnaire

A self-report questionnaire was administered to measure risky sexual behaviour. Participants were asked to report the age at which they first had sexual intercourse, and the number of people with whom they have had sexual intercourse during their lifetime (both continuous variables). Participants then reported the nature of the encounter with their two most recent sexual partners, including (i) their relationship with their partner at the time of the encounter (a response that they had just met this person for the first time was considered to be ‘high risk’) and (ii) whether or not they used a condom during the encounter. Finally, participants reported whether they had ever been diagnosed with an STI.

### 2.3. Procedure

Ethical approval was granted by the relevant local ethical review board. All testing sessions took place at either 1:00 pm or 2:00 pm so that any influence of the cortisol awakening response (Fries, Dettenborn, & Kirschbaum, 2009) could be avoided and because cortisol reactivity tends to be greater in the afternoon (Kirschbaum & Hellhammer, 1989). Participants were screened for the exclusion criteria and basic demographic information was collected, before the first saliva sample was obtained. The researcher then provided instructions for the TSST, before a second saliva sample was obtained. The participant was given five minutes to prepare for the public speaking component of the TSST, after which the researcher returned, collected a third saliva sample and directed the participant to another room where they completed the TSST. After a further 20-minute period, the final saliva sample was collected and the participant then completed the sexual behaviour questionnaire. The overall testing period lasted between 50 and 60 minutes.

### 2.4. Data analysis

For the continuous risky behaviours (age at first sexual intercourse and number of lifetime intercourse partners), a correlation analysis was conducted between the sexual behaviour and area under the cortisol response curve (AUC) with respect to increase. This conceptualisation of AUC enables the collapse of multivariate data (each sampling time-point) into a single value which reflects both the magnitude of the response and change over time. AUC with respect to increase is determined on the basis of increases from baseline, and is thought to be particularly suggestive of the sensitivity of the cortisol response to stress. Larger values reflect a greater magnitude in the response to stress over time (Fekedulegn et al., 2007). For the dichotomous risky behaviours (condom use, STIs, intercourse with a person just met), change from baseline values were obtained for each of the cortisol sampling time-points, and data were subjected to a 2 (risk; high, low) × 4 time (post-instructions, post-preparation, immediately post-TSST, 20-minute post-TSST) mixed analysis of variance (ANOVA), with risk being a between-subjects factor and time being a repeated measures factor. The dependent variable was the cortisol value at each time-point. Significant risk × time interaction effects and main effects of time were followed up with Bonferroni-adjusted pairwise comparisons. The treatment of the dichotomous risky behaviours using an ANOVA enables the conceptualisation of the precise change in cortisol at each measurement time-point. It is not possible to analyse the continuous outcomes in this way without artificially imposing an arbitrary cut-off value on these variables. On this basis, values for the continuous risk-taking behaviour variables are correlated against AUC, while the dichotomous risk-taking behaviour variables are analysed using ANOVA from which the change in cortisol at each measurement time-point can be ascertained.

## 3. Results

### 3.1. Correlations between age at first sexual intercourse, number of lifetime intercourse partners and cortisol reactivity

The mean age at first sexual intercourse in this sample was 17.3 years (SD = 2.5). The mean number of lifetime intercourse partners was 7.8 (SD = 8.9). No significant relationship was observed between cortisol reactivity (AUC) and (i) age at first sexual intercourse, *r* = −0.07, *p* = .74; or (ii) number of lifetime intercourse partners, *r* = 0.03, *p* = .87.

### 3.2. Condom use, STIs and intercourse with someone just met

A total of 22 participants (84.6%) reported that they used a condom with each of their last two intercourse partners, while the four participants (15.4%) who did not use a condom with at least one of these partners were classified as high risk. On cortisol reactivity, the time × risk interaction effect was nonsignificant, *F* (3, 22) = 0.28, *p* = .84, with the effect size being small, partial *η* ^2^ = 0.04. The main effects of time, *F* (3, 22) = 2.28, *p* = .11, partial *η* ^2^ = 0.24, and risk, *F* (1, 24) = 0.01, *p* = .93, partial *η* ^2^ < 0.01, were also nonsignificant.

Only two participants (7.7%) reported that they had previously been diagnosed with an STI. For this reason, it was decided not to compare cortisol reactivity between those participants who reported having been diagnosed with an STI against those who had not.

**Figure 1.**
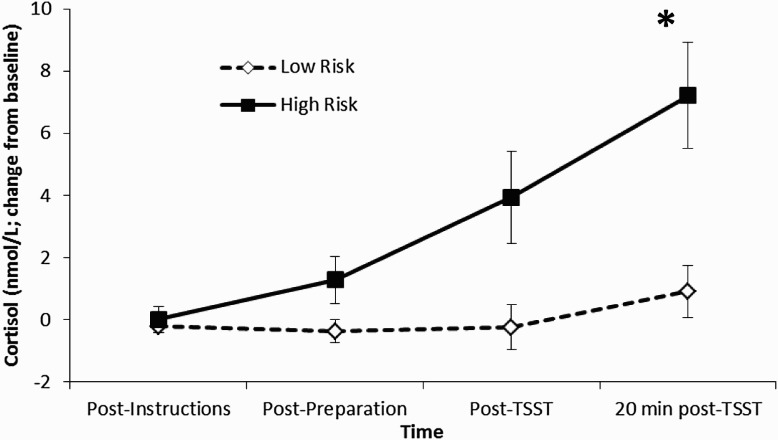
Cortisol reactivity (change from baseline) to the TSST at each sampling time-point for those participants who reported that one of their most two recent sexual partners was someone whom they had just met (high risk; *n* = 5) compared to those participants who did not report that one of their most two recent sexual partners was someone whom they had just met (low risk; *n* = 21). Bonferroni-adjusted pairwise comparisons revealed that high risk participants showed a significantly greater cortisol response 20-minute following the TSST.

Five participants (19.2%) reported that at least one of their last two sexual partners was someone whom they had just met for the first time. On cortisol reactivity, the time × risk interaction effect was significant, *F* (3, 22) = 3.30, *p* < .05, with the effect size being moderate, partial *η* ^2^ = 0.31. Bonferroni-adjusted pairwise comparisons (adjusted *α* = 0.0125) revealed that the cortisol value relative to baseline at the 20-minute post-TSST time-point was higher for the high risk, relative to the low risk group (*p* = .003), but was nonsignificant for the other sampling time-points. The main effect of time, *F* (3, 22) = 6.62, *p* < .01, partial *η* ^2^ = 0.48, was also significant, with the 20-minute post-TSST cortisol value being significantly higher than the other three sampling time-points (all Bonferroni adjusted *p* < .017). Finally, the main effect of risk was also significant, *F* (1, 24) = 8.92, *p* < .01, partial *η* ^2^ = 0.27 (see Figure 1).

## 4. Discussion

The most notable finding from this study was that individuals who reported that at least one of their two previous sexual partners was a person whom they had just met demonstrated significantly greater cortisol reactivity to an acute stressor involving psychosocial evaluation. On this basis, it appears that reactivity of the HPA axis to stress is greater in individuals who engage in high risk sexual behaviour. However, similar to previous research which has addressed the question of the relationship between cortisol reactivity and sexual risk taking (Halpern et al., 2002), we did not observe this effect for *all* of the sexual risk-taking behaviours which we investigated.

To the best of our knowledge, this study is only the second report in the literature of an increased cortisol response to stress in individuals engaged in risky sexual behaviour. The previous study which reported a similar observation found that individuals who were classified as ‘reactors’ to stress tasks involving public speaking and a mock dating interview were less likely to use condoms (Halpern et al., 2002). In interpreting this finding, the authors suggested that individuals who are prone to greater psychobiological stress reactivity may be less likely to use condoms due to the stressful nature of condom use negotiation (Halpern et al., 2002). However, this same argument cannot be used to support the findings of this study. We did not replicate the finding of Halpern and colleagues, in that that cortisol reactivity to stress was not found to be greater in individuals who reported that they did not use a condom with at least one of their last two sexual partners, but we did find that cortisol reactivity was greater in individuals who reported that they had just met either of their past two sexual partners at the time of the intercourse encounter. On initial consideration, this finding seems counterintuitive, as one might expect that individuals with a disposition towards greater psychobiological responding to stress would avoid the potentially stressful experience of engaging in sexual intercourse with a person whom they had just met.

Dispositional sensation seeking is one factor that could potentially explain our finding that participants who reported that one of their two previous intercourse partners was someone whom they had just met showed greater cortisol reactivity to stress. Sensation seeking is highly predictive of the propensity to engage in risk-taking behaviour (Donohew et al., 2000), and it is reasonable to assume that individuals high in dispositional sensation seeking may actively seek a state of hormonally induced physiological arousal by engaging in risk-taking behaviour. However, some studies have found that cortisol reactivity is in fact lower in high sensation seeking individuals (Roberti, 2004) rendering this explanation of the findings unlikely. However, heightened cortisol responses to the TSST have been associated with greater risk taking on a laboratory task, with the authors concluding that higher post-stress cortisol levels could be related to greater feelings of euphoria, which may adversely influence decision-making processes (van den Bos, Harteveld, & Stoop, 2009). Alternatively, while we did not ask participants whether they were under the influence of drugs or alcohol during the encounters in which they had intercourse with a person whom they had just met, such encounters are more likely to occur following the ingestion of alcohol (Anderson & Mathieu, 1996). Therefore, a further explanation for the observations reported here could be that individuals with stress-related HPA axis dysregulation could be more prone to heavy alcohol consumption (Thayer, Hall, Sollers Iii, & Fischer, 2006), which increases the likelihood of sexual activity with a partner whom they do not know. However, while previous research relating to heavy alcohol use in healthy individuals and cortisol reactivity to stress is limited, it has been reported previously that the cortisol response to stress is blunted in alcohol-dependent individuals (Lovallo, Dickensheets, Myers, Thomas, & Nixon, 2000), which casts doubt over alcohol as an explanation for this study findings.

In a more general sense, one proposed model of risk taking purports that later life risk taking has early life origins, whereby early life stressors lead to both dysregulation of the HPA axis and later life risk taking (Romer, 2010). The model purports that exposure to childhood stressors such as physical abuse, emotional abuse, exposure to violence and parental substance abuse can lead to later life risk-taking behaviours such as substance abuse, while sexual abuse in particular is a predictor of sexual risk-taking behaviours including earlier age at first sexual intercourse and unplanned pregnancy. This model of risk taking provides an epigenetic explanation for heightened reactivity to stress in individuals exposed to early life stress, suggesting that greater stress exposure in childhood ‘silences’ genes which regulate HPA axis function. Therefore, the possibility that early life stress exposure may be the mechanism underpinning the relationship between cortisol reactivity and sexual risk taking cannot be discounted. Future studies should adopt a prospective design to investigate the influence of early life stress exposure on later life reactivity of the HPA axis and sexual risk-taking behaviour in order to better understand the role of early life stress as a risk factor for such behaviours. Additionally, Romer's (2010) model also cites animal research to suggest that dysregulation of the HPA axis adversely affects the developing brain. Exposure to early life stress and the resultant dysregulation of the HPA axis is known to impact upon the optimal development of key brain structures such as the hippocampus, amygdala and prefrontal cortex, which play a role in such cognitive functions as memory, executive functioning and emotion regulation (Lupien, McEwen, Gunnar, & Heim, 2009). It is possible that impairments in these cognitive domains could have implications in terms of capacity for optimal decision-making processes, which may predispose an individual to engage in risk-taking behaviour. These mechanisms provide an interesting possible explanation for this study findings. However, it is important to note that early life stress and neurocognitive development were not measured in this study, so implication of their involvement in the relationship between HPA reactivity to stress and sexual risk taking is speculative. Nevertheless, this framework provides an intriguing hypothesis which warrants investigation in future studies.

In this study, we investigated the relationship between engagement with five sexual risk-taking behaviours and cortisol reactivity to psychosocial stress. A novel finding of our study was that individuals who reported that at least one of their previous two sexual partners was someone whom they had just met exhibited greater cortisol reactivity to the TSST. However, previous findings which suggested a relationship between cortisol reactivity to stress and sexual risk-taking behaviours including age at first intercourse (Brody, 2002), condom use (Halpern et al., 2002) and number of lifetime intercourse partners (Halpern et al., 2002) were not replicated here. Further, an aim of this study was to investigate the relationship between STI diagnosis and cortisol reactivity to stress, but an insufficient number of participants in our sample reported having been diagnosed with an STI to enable a meaningful analysis. What remains unclear is whether engagement with specific sexual risk-taking behaviours is related to reactivity of the HPA axis to psychosocial stress, or whether sexual risk taking more generally is relevant in this context. In the preceding paragraphs, we have suggested a number of mechanisms by which dysregulation of the HPA axis and atypical cortisol responses to acute stress may predict the engagement with specific sexual risk-taking behaviours. However, further studies employing very large sample sizes are required in order to determine a profile of risk-taking behaviours which may be influenced by HPA axis function, and to establish causality in this relationship.

The findings of this study should be considered in light of its limitations. As mentioned above, the sample size of 26 individuals is relatively small, and it is of course possible that the one significant finding in this study is attributable to chance. In addition, the majority of individuals who took part in this study were university students; therefore, the relative selectiveness of this sample may bias the generalisability of the study findings. Further, only a small proportion of the sample reported engagement with the risk-taking behaviours of interest. There were an insufficient number of participants who reported that they had been diagnosed with an STI to investigate cortisol reactivity to stress in these individuals. This study may not have been sufficiently powered to detect differences in cortisol reactivity between risk takers and non-risk takers with respect to some of the sexual behaviours under investigation. While this study findings are informative with respect to the identification of a potentially important link between reactivity of the HPA axis and sexual risk taking, replication of these findings in a substantially larger sample is needed. A larger sample size would enable conclusions to be drawn with respect to whether participants exhibit a profile of risky behaviours which amplifies the risk of adverse consequences (e.g. an individual engaging in sexual intercourse with a partner whom they had just met could be considered to be at a substantially reduced risk of adverse sexual health or unplanned pregnancy if a condom and other contraceptive methods were employed during this encounter). Further work should also control statistically for other factors which could confound the findings, such as SES. The small sample size also limited our capacity in this study to account for situations in which a lack of condom use could be considered relatively low risk, such as individuals in an exclusive, long-term relationship, or individuals trying to conceive. These limitations notwithstanding, this study provides valuable preliminary evidence for a link between cortisol reactivity to stress and sexual risk-taking behaviours which warrants further investigation in a larger study. A further limitation is that participants were screened for potentially sensitive exclusion criteria immediately prior to providing the first saliva sample and there is a small possibility that this may have been sufficiently distressing for some individuals to evoke a change in cortisol levels.

In this study, we found that individuals who reported that least one of their past two intercourse partners was someone whom they had just met exhibited heightened cortisol reactivity to psychosocial stress. This finding supports a previous report in the literature that individuals who exhibited cortisol reactivity to stress reported greater sexual risk-taking behaviour in the form of less frequent condom use (Halpern et al., 2002). Future work should investigate the mechanisms underpinning the relationship between HPA axis function and sexual risk-taking behaviour. In accordance with the findings of van den Bos et al. (2009), we suggest that the euphoria associated with heightened stress reactivity may influence sexual risk taking by diminishing decision-making processes. We further argue that deficits in decision-making relating to sexual health behaviours could result from early life stress, which dysregulates HPA function and adversely impacts upon the optimal development of key brain areas involved in cognitive and emotional processes relevant to decision-making.
